# A Counting Stroop Functional Magnetic Resonance Imaging Study on the Effects of ORADUR-Methylphenidate in Drug-Naive Children with Attention-Deficit/Hyperactivity Disorder

**DOI:** 10.1089/cap.2022.0024

**Published:** 2022-11-15

**Authors:** Chi-Yung Shang, Tai-Li Chou, Cheng-Yu Hsieh, Susan Shur-Fen Gau

**Affiliations:** ^1^ Department of Psychiatry, National Taiwan University Hospital and College of Medicine, Taipei, Taiwan.; ^2^ Department of Psychology, National Taiwan University, Taipei, Taiwan.; ^3^ Graduate Institute of Brain and Mind Sciences, National Taiwan University, Taipei, Taiwan.

**Keywords:** attention-deficit/hyperactivity disorder, methylphenidate, functional brain imaging, counting Stroop task, Rapid Visual Information Processing, Conners' Continuous Performance Test

## Abstract

**Objective:**

Methylphenidate is effective in reducing the clinical symptoms of patients with attention-deficit/hyperactivity disorder (ADHD). ORADUR^®^-methylphenidate is a new extended-release preparation of methylphenidate. This study aimed at identifying brain regions with activation changes and their correlations with neuropsychological functions after treatment with ORADUR-methylphenidate in children with ADHD.

**Methods:**

We recruited drug-naive children with ADHD and age- and sex-matched typically developing (TD) children. They were all scanned with the functional magnetic resonance imaging (fMRI) during the counting Stroop task at baseline, and those with ADHD had the second fMRI assessment after 8-week treatment with ORADUR-methylphenidate. The Rapid Visual Information Processing (RVP) and Conners' Continuous Performance Test (CCPT) were used to assess the attention performance of the ADHD (before and after treatment) and TD groups.

**Results:**

ORADUR-methylphenidate significantly decreased inattention (Cohen *d* = 2.17) and hyperactivity-impulsivity (Cohen *d* = 0.98) symptoms. We found less activation in the right inferior frontal gyrus (rIFG) in the pre-treatment ADHD children than TD children and greater treatment-induced activation in the dorsal anterior cingulate cortex (dACC) and the right dorsolateral prefrontal cortex (rDLPFC). There was no significant difference between the post-treatment ADHD and TD groups. However, the treatment-related activations in the dACC, rDLPFC, and rIFG were significantly correlated with CCPT and RVP measures.

**Conclusions:**

Our findings indicated that ORADUR-methylphenidate increased brain activations in the dACC, rDLPFC, and rIFG in children with ADHD, associated with improved focused attention, reduced impulsivity, and enhanced inhibition control. Activities of these brain regions might be biomarkers for the treatment effectiveness of methylphenidate for ADHD.

**Clinical Trials Registration:**

ClinicalTrials.gov number, NCT02450890

## Introduction

Attention-deficit/hyperactivity disorder (ADHD) is a neurodevelopmental disorder with significant functional impairments mediated by executive dysfunctions (Gau et al. 2015). Current pharmacotherapy in ADHD is based on the hypothesis of dopaminergic and noradrenergic dysregulation (Cortese 2020). Although methylphenidate, a dopamine and noradrenaline reuptake inhibitor (Faraone 2018), has been widely used in treating ADHD, the impact of methylphenidate on neural networks is incompletely understood.

Neuropsychological dysfunctions have been identified as one of the major targets in treating ADHD (Wu et al. 2021). Previous studies have demonstrated that methylphenidate is not only effective in reducing the core symptoms of ADHD but also improves a wide range of neuropsychological functions, including sustained attention (Bedard et al. 2015), impulsivity (Chou et al. 2015), and response inhibition (Broyd et al. 2005).

Approaches with task-based functional magnetic resonance imaging (fMRI) have been used to identify the effects of methylphenidate on brain activation in children with ADHD. For example, acute administration of methylphenidate enhanced activations in the right ventrolateral prefrontal region in boys with ADHD, which was correlated with improvement in inhibitory control (Cubillo et al. 2014). In addition, chronic administration of methylphenidate improved the severity of ADHD symptoms related to decreased activations in the left anterior cingulate, left supplementary motor area, right inferior frontal gyrus (rIFG), and bilateral posterior cingulate during a response inhibition task (Schulz et al. 2012).

Our previous work showed that chronic administration of methylphenidate enhanced activations in the inferior frontal gyrus during a counting Stroop task (Chou et al. 2015). However, these fMRI studies were heterogeneous in methodology and thus demonstrated a degree of inconsistency in brain activation or deactivation (Zimmer 2017).

Several types of once-a-daily methylphenidate formulations with various pharmacokinetic profiles have been developed. Different pharmacokinetic profiles might be associated with variations in the effects of methylphenidate on the brain (Spencer et al. 2010). Our recent work has demonstrated the effectiveness of a new once-a-day product of methylphenidate, ORADUR^®^-methylphenidate, in reducing the clinical symptoms of children with ADHD (Huang et al. 2020).

The aim of the present study is to assess the effectiveness of ORADUR-methylphenidate in improving focused attention and inhibitory control by using the fMRI with the counting Stroop task in children with ADHD. Given that the rIFG (Zhang et al. 2017; Tremblay et al. 2020), the right dorsolateral prefrontal cortex (rDLPFC) (Rubia et al. 2009b; McNeill et al. 2018), and the dorsal anterior cingulate cortex (dACC) (Chou et al. 2015; Fan et al. 2018) are crucial for inhibitory control and attentional processing, we hypothesized that ORADUR-methylphenidate would upregulate functional activations in these brain regions.

In addition, the Rapid Visual Information Processing (RVP) and the Conners' Continuous Performance Test (CCPT) outside the scanner was used to examine whether the treatment-related changes in brain activations observed during fMRI were associated with the treatment-related changes in the neuropsychological measures.

## Methods

### Participants

We recruited 49 drug-naive children diagnosed with ADHD according to *Diagnostic and Statistical Manual of Mental Disorders, Fifth Edition* (DSM-5) (American Psychiatric Association, 2013) from the Department of Psychiatry, National Taiwan University Hospital (NTUH), Taipei, Taiwan. The parents of all the participants were interviewed with the Mandarin version of the Schedule for Affective Disorders and Schizophrenia for School-Age Children-Epidemiological Version (K-SADS-E) for DSM-5 (Chen et al. 2017) by the corresponding author (S.S.-F.G.) to confirm the clinical diagnosis of ADHD and to exclude other psychiatric disorders. In addition, all participants received an intelligence assessment using Wechsler Intelligence Scale for Children.

Participants were excluded if they had a history of major psychiatric disorders, including schizophrenia, schizoaffective disorder, affective disorders, substance abuse, or pervasive developmental disorder; a history of seizure; a serious medical illness; Full-Scale IQ (FIQ) score <80; or if they had any prior or current psychotropic medication. Written informed consent was obtained from all participants' parents or legal representatives before performing any protocol-specific procedure.

The Research Ethics Committee at NTUH approved the informed consent procedures before implementing the current study (approval number: 201412007MSB; ClinicalTrials.gov number, NCT02450890).

Another 28 typically developing (TD) children with matched age and sex were enrolled in the control group. Based on K-SADS-E interviews, they had no DSM-5 psychiatric disorder in their lifetime (Chen et al. 2019). Those who had any neurological or medical conditions, who took any psychotropic medication, or whose FIQ scores were <80 were excluded.

The participants with ADHD began medications after visit 1 with ORADUR-methylphenidate. For all enrolled subjects, the initial dosing period was 22 mg per day for 1 week. Then, the investigators titrated the medication dosage based on the clinical response and adverse effects.

The counting Stroop task was performed during the MRI scan at baseline before treatment initiation and week 8. To achieve maximum efficacy with considering the pharmacokinetics of ORADUR-methylphenidate, participants with ADHD were required to take the medication in the morning 2–4 hours before the second fMRI assessment. In addition, the participants performed the RVP and the CCPT outside the MRI scanner at baseline and week 8.

### Behavioral and neuropsychological measurements

#### ADHD symptom severity

We assessed the clinical severity of all the ADHD participants with the number of ADHD symptoms according to DSM-5 criteria by using the ADHD supplement of K-SADS-E, including the two symptom domains of inattention and hyperactivity/impulsivity (Chen et al. 2017).

#### Clinical Global Impression-ADHD Severity Scale

The Clinical Global Impression-ADHD Severity Scale (CGI-ADHD-S) was a single-item rating of the clinician's assessment of the global severity of ADHD symptoms concerning the clinician's experience with other patients with ADHD. The Chinese version of the CGI-ADHD-S has been widely used in ADHD treatment studies in Taiwan (Gau et al. 2007; Chang et al. 2021).

#### Rapid Visual Information Processing

The sustained visual attention of the subjects was assessed by the RVP (Sahakian et al. 1989). In random order, digits appeared one at a time (100 digits/min) in the center of the screen. Subjects had to detect three target sequences (3–5–7, 2–4–6, 4–6–8) and respond using a press pad when seeing the last number (7, 6, and 8, respectively). Total hits represented the number of occasions that the subjects correctly responded to the target sequences. In contrast, total misses represented the number of events they failed to respond to the target sequence.

Four indices reported included (1) probability of hits (*h*): total hits divided by the sum of total hits and total misses; (2) probability of false alarms (*f*): total false alarms divided by the sum of total false alarms and total correct rejections; (3) A′: (0.5 + [(*h* − *f*) + (*h* − *f*)^2^]/[4 × *h* × (1 − *f*)]): a signal detection measure of sensitivity to the target, regardless of response tendency; and (4) mean latency: mean time taken to respond in the correct responses. The RVP has been used in many ADHD research to assess sustained attention and inhibition control (Fan et al. 2018; Gau and Huang 2014; Shang et al. 2021).

#### Conners' Continuous Performance Test

The CCPT was a computerized task (Conners and Staff, 2000), widely used to measure focused attention and inhibitory control in patients with ADHD (Lin et al. 2013; Wu et al. 2014; Cheng et al. 2020). The subjects were required to respond when letters appeared on the screen except for the letter X. Two indices were reported, including response style and perseveration. Response style was defined as a function of the ratio of hit target stimuli to hit non-target ones.

The perseveration was defined as the responses with a reaction time <100 ms related to impairment in inhibitory control. The *t*-scores of the response style and perseveration were presented, defined as multiplying the *z*-score by 10 and adding 50, with a mean of 50 and a standard deviation of 10.

#### Functional activation task

A counting Stroop task was used to investigate the differences in the neural substrates of inhibitory control between these two groups of participants. In this task, experimental stimuli were classified into three conditions: congruent, incongruent, and control conditions (Supplementary Fig. S1). In the congruent condition, the number of words was consistent with the meaning of the word such as “one,” “two,” “three,” or “four,” whereas the number of words was inconsistent with the meaning of the word in the incongruent condition.

In the control condition, the meaning of the words did not give any clue to the number. The number of syllables, visual complexity (strokes per word), and frequency of all the words across the three conditions were well matched. During fMRI scans, participants were instructed to report the number of words (one to four) by pressing a button, regardless of the word meaning, during fMRI scans. This counting Stroop task has been used in our previous imaging studies (Fan et al. 2014, 2017, 2018; Shang et al. 2018).

### MRI image acquisition

We acquired images by employing a 3T Siemens Tim-Trio scanner with a 32-channel head coil. Subjects looked at the visual stimuli projected onto a screen via a mirror attached to the head coil. Each subject performed two 2.8-minutes functional runs. Eighty-five image volumes were acquired in each run using echo-planar imaging to detect the BOLD (blood oxygenation level-dependent) signal.

Functional images were interspersed from bottom to top and collected parallel to the AC–PC plane. The scanning has the following parameters: repetition time (TR) = 2000 ms; echo time (TE) = 24 ms; flip angle = 90°; matrix size = 64 × 64; field of view = 25.6 cm; slice thickness = 3 mm; and number of slices = 34. A high-resolution, T1-weighted three-dimensional image was also acquired (Magnetization Prepared Rapid Gradient Echo, MP-RAGE; TR = 2300 ms; TE = 2.98 ms; flip angle = 9°; matrix size = 256 × 256; field of view = 25.6 cm; slice thickness = 1 mm).

The orientation of the 3D image was identical to the functional slices. The task stimuli were administered in a pseudorandom order for all participants to optimize the event-related design.

### Image and statistical analysis

The percentage was used for categorical variables, and mean scores and standard deviation are presented for continuous variables. We conducted a series of paired *t*-tests to examine the treatment-related changes in the number of DSM-5 ADHD symptoms, CGI-ADHD-S, RVP, CCPT, and counting Stroop at week 8 compared with baseline. To avoid the multiple comparison problem, we decided on the significance of each test by employing the Benjamini–Hochberg procedure (Benjamini and Hochberg, 1995), with a false discovery rate set to be 5%.

Cohen's *d* was used to compute effect sizes on the inter-session variance for the comparisons between baseline and week 12, with small (Cohen's *d*, 0.2–0.5), medium (Cohen's *d*, 0.5–0.8), and large (Cohen's *d*, ≥0.8) effect sizes. In addition, we conducted two-way ANOVA to examine the behavioral improvements in the counting Stroop task (i.e., accuracy, reaction time). Family-wise error (FWE) rate was controlled at 5%.

Imaging data analysis was performed using Statistical Parametric Mapping (SPM). The functional images were corrected for the differences in slice-acquisition time to the middle volume and were realigned to the first volume in the scanning session using affine transformations. The exclusion criteria for the motion were 3 mm for displacement and 3° for rotations (Supplementary Table S2). Co-registered images were normalized to the Montreal Neurological Institute (MNI) average template. Statistical analyses were calculated on the smoothed data (10 mm isotropic Gaussian kernel, the concept of the kernel is defined as the shape of function to calculate the weighted average of each data point with its neighboring data points), with a high pass filter (128 seconds cutoff period) to remove low-frequency artifacts.

Data from each participant were entered into a general linear model using an event-related analysis procedure. Stimuli were treated as individual events for analysis and modeled using a canonical Hemodynamic Response Function (HRF). Parameter estimates from contrasts of the canonical HRF in single-subject models were entered into random-effects analysis using one-sample *t*-tests across all participants to determine whether activation during a contrast was significant (i.e., parameter estimates were reliably greater than 0) in a whole-brain analysis.

There were three types of events: congruent, incongruent, and control in the counting Stroop task. The present study used the incongruent condition versus congruent condition to explore the neural correlates of inhibitory control within each group (ADHD pre-treatment, ADHD post-treatment, TD). All reported areas of activation were significant using *p* < 0.005 uncorrected at the voxel size larger than 10 in a whole-brain analysis. Comparisons between groups were also examined, with all reported areas of activation significant using *p* < 0.005 uncorrected at the voxel size larger than 10 in a whole-brain analysis.

For the significant voxels selected by our hypothesis, the areas of activation were also significant using *p* < 0.05 FWE corrected with an anatomical mask from the WFU PickAtlas toolbox for SPM8, that is., the rIFG, rDLPFC, and dACC.

To examine the correlations between the changes in neuropsychological performances and the changes in brain activation, those clusters showing significant effects of methylphenidate were identified as Regions of Interest (ROIs). We then extracted the beta value (signal intensity) for these ROIs in the ADHD group at pre-treatment and post-treatment for partial correlations with the measures of RVP and CCPT, adjusting for ADHD symptoms (i.e., CGI-ADHD-S, DSM Criteria-inattention, and DSM Criteria-hyperactivity/impulsivity). All reported results were significant at *p* < 0.05 level.

## Results

### Clinical results

Supplementary Figure S2 shows the flowchart of the study procedure. Of the 49 children with ADHD recruited in the present study, participants dropped out because of adverse effects (*n* = 5) and personal reasons (*n* = 1). The imaging data of another 15 participants were not included in the final analyses due to abnormality in structural MRI (*n* = 1) and poor imaging quality (*n* = 14), with 28 participants in the final ADHD group. There were no significant differences in age, sex, and FIQ between the ADHD and TD groups (all *p*-values >0.05, Supplementary Table S1). Table 1 summarizes the pre-treatment and post-treatment clinical symptoms in the ADHD group.

**Table 1.** The Clinical Symptoms and Neuropsychological Performances on Rapid Visual Information Processing and Conners' Continuous Performance Test for the Attention-Deficit/Hyperactivity Disorder Group at the Pre-treatment and the Post-treatment Periods

	Pre-treatment	Post-treatment	t-Statistics,* t *(27)	*p*	Cohen's d
CGI-ADHD-S	5.86 (0.45)	3.54 (0.88)	−12.04	<0.001^**^	2.32
DSM ADHD Criteria Symptom					
Inattention	8.29 (0.85)	2.68 (2.25)	−11.47	<0.001^**^	2.17
Hyperactivity-impulsivity	5.07 (2.76)	1.79 (1.95)	−5.20	<0.001^**^	0.98
RVP					
A′ (target sensitivity)	0.82 (0.07)	0.81 (0.07)	0.35	0.73	0.07
Mean latency	628 (140)	585 (166)	−1.10	0.28	0.21
Probability of hit	0.46 (0.21)	0.41 (0.17)	0.85	0.41	0.16
Probability of false alarm	0.08 (0.10)	0.06 (0.09)	0.85	0.40	0.16
CCPT					
Response style	47.44 (13.16)	53.82 (18.36)	1.71	0.10	0.32
Perseveration	50.37 (9.93)	58.72 (17.27)	2.71	0.01^*^	0.51

^*^ *p* < 0.05; ^**^p < 0.001.

ADHD, attention-deficit/hyperactivity disorder; CCPT, Conners' Continuous Performance Test; CGI-ADHD-S, Clinical Global Impression-ADHD Severity Scale; RVP, Rapid Visual Information Processing.

We found a significant improvement in the CGI-ADHD-S, indicative of overall symptom severity (*t* = −12.04, *p* < 0.001, Cohen's *d* = 2.32). We also found significant reductions in the number of clinical symptoms based on DSM-5 ADHD Criteria, in terms of both inattention (*t* = −11.47, *p* < 0.001, Cohen's *d* = 2.17) and hyperactivity-impulsivity (*t* = −5.20, *p* < 0.001, Cohen's *d* = 0.98).

### Neuropsychological results

Table 1 summarizes the performance on the RVP task of the CANTAB and the CCPT. Regarding the performance on the RVP, a nonsignificant post-treatment minus pre-treatment difference was shown in the A′ (target sensitivity), *t*(27) = 0.35, *p* = 0.73, Cohen's *d* = 0.07 (Supplementary Fig. S3a), and in the probability of hit, *t*(27) = 0.85, *p* = 0. 41, Cohen's *d* = 0.16 (Supplementary Fig. S3b). Regarding the performance on the CCPT, a significant post-treatment minus pre-treatment difference was revealed in the perseveration (transformed into *T* score), *t*(27) = 2.98, *p* = 0.006, Cohen's *d* = 0.56, with a higher value of perseveration at post-treatment relative to pre-treatment (Supplementary Fig. S3c).

### Behavioral results

Supplementary Table S3 presents the reaction time and accuracy of the counting Stroop task for the ADHD group pre- and post-treatment. First, regarding the accuracy, no interaction effect was significant, *F*(2, 54) = 2.01, Mean Squared Error (MSE) < 0.01, *p* = 0.155, $\eta_{G}^{2}$ = 0.01. The main effect of treatment was also not significant, *F*(1, 27) = 0.93, MSE = 0.01, *p* = 0.345, $\eta_{G}^{2}$ = 0.01.

However, the main impact of the condition was significant, *F*(2, 54) = 4.39, MSE < 0.01, *p* = 0.020, $\eta_{G}^{2}$ = 0.03, with higher accuracy for both the congruent and control conditions relative to the incongruent condition. Second, regarding reaction time, no interaction effect was significant as well, *F*(2, 54) = 1.03, MSE = 8333.9, *p* = 0.344, $\eta_{G}^{2}$ = 0.04. However, the main effect of treatment was significant, *F*(1, 27) = 9.88, MSE = 27767, *p* = 0.004, $\eta_{G}^{2}$ = 0.03, with a faster reaction time post-treatment relative to pre-treatment.

The main effect of the condition was also significant, *F*(2, 54) = 10.99, MSE = 6910, *p* < 0.001, $\eta_{G}^{2}$ = 0.014, with a faster reaction time for both the congruent and control conditions relative to the incongruent condition.

### Neuroimaging results

**FIG. 1.**
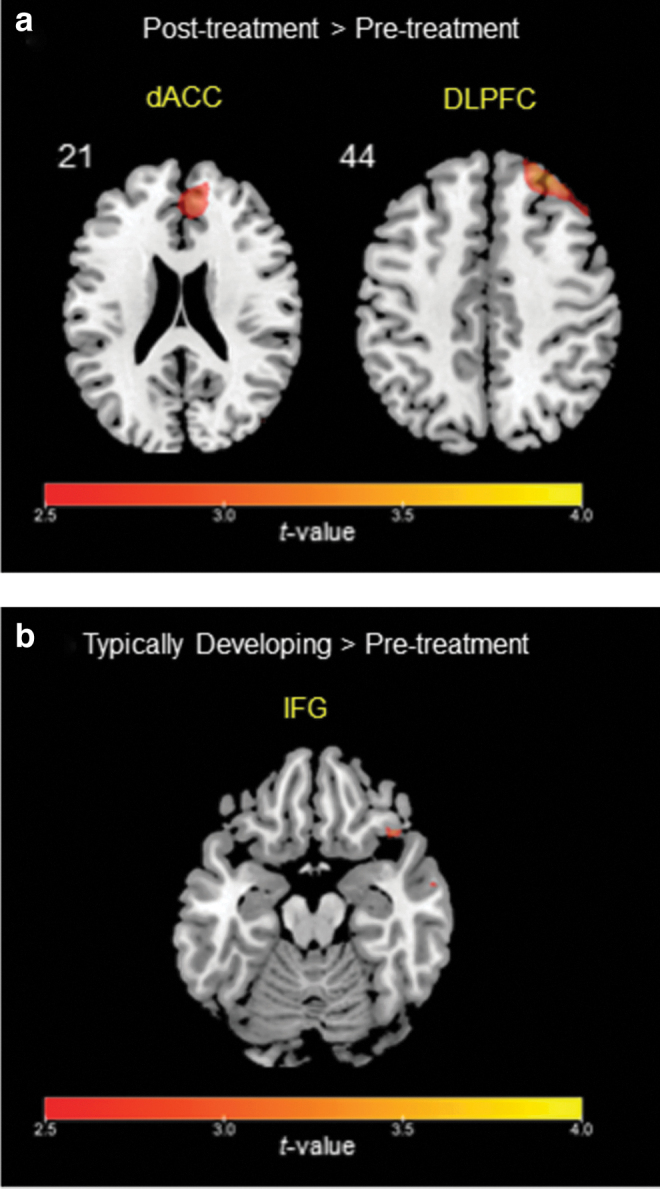
**(a)** Greater activation in the dACC and the rDLPFC from pre-treatment to post-treatment in children with ADHD for the incongruent versus congruent condition. **(b)** Greater activation in the rIFG in the TD group relative to the ADHD group at pre-treatment for the incongruent versus congruent condition. Reported areas of activation indicate the significance using uncorrected *p* < 0.005, a voxel size larger than 10. ADHD, attention-deficit/hyperactivity disorder; dACC, dorsal anterior cingulate cortex; rDLPFC, right dorsolateral prefrontal cortex; rIFG, right inferior frontal gyrus; TD, typically developing.

In the present study, the contrast of the incongruent condition versus congruent condition was used to explore the neural correlates of inhibitory control within each group (ADHD pre-treatment, ADHD post-treatment, TD, see Tables 2 and 3). Compared with the pre-treatment, more activation was found in the dACC and rDLPFC at the post-treatment in children with ADHD (Fig. 1a and Table 2). A significant difference in the rIFG was also found at a lower threshold between the pre-treatment and post-treatment in the ADHD group (Table 2). Moreover, compared with the TD group, the ADHD group showed pre-treatment hypo-activation in the rIFG (Fig. 1b).

**Table 2.** Activation of Cortical Regions for the Incongruent Versus Congruent Condition for the Attention-Deficit/Hyperactivity Disorder Group at the Pre-treatment and Post-treatment, and the Comparison Between the Two Time Points

Cortical regions	H	BA	Voxels	*Z* test	MNI coordinates		
					*x*	*y*	*z*
ADHD group							
Pre-treatment							
Supplementary motor area	R	6	1462	4.04	4	−4	58
Supplementary motor area	L			3.32	−6	−6	64
Inferior parietal lobule	L	40	1322	3.99	−60	−24	30
Precentral gyrus	R	4	687	3.71	36	−24	60
Inferior frontal gyrus	L	47	34	3.34	−50	30	−10
Post-treatment							
Dorsolateral prefrontal cortex^a^	R	9	448	3.21	30	36	48
Inferior frontal gyrus^b^	R	47	226	3.19	44	34	−16
Dorsal anterior cingulate cortex^b^	R	24	146	3.16	4	18	26
Pre-treatment>Post-treatment							
Postcentral gyrus	L	2	5496	4.01	−50	−34	58
Superior temporal gyrus	L	22		3.75	−38	−62	14
Inferior parietal lobule	L	40		3.63	−34	−38	46
Middle temporal gyrus	L	39		3.62	−36	−64	18
Supplementary motor area	L	6		3.32	4	−14	66
Post-treatment>Pre-treatment							
Angular gyrus	R	39	190	3.55	44	−74	34
Dorsolateral prefrontal cortex^b^	R	9	961	3.37	20	54	34
Dorsal anterior cingulate cortex^b^	R	9		2.93	8	42	28
Inferior frontal gyrus^c^	R	47	58	2.74	30	28	−16

Coordinates of activation peak(s) within a region based on a *z* test are given in the MNI stereotactic space (*x, y, z*). All reported regions were *p* < 0.005 uncorrected with a voxel size larger than or equal to 10.

^a^ *p* < 0.10 for FWE corrected with the use of an anatomical mask.

^b^ *p* < 0.05 for FWE corrected with the use of an anatomical mask.

^c^ *p* < 0.01 uncorrected with a voxel size larger than or equal to 10.

ADHD, attention-deficit/hyperactivity disorder; BA, Brodmann's area; FWE, family-wise error; H, hemisphere, L, left; MNI, Montreal Neurological Institute; R, right.

**Table 3.** Activation of Cortical Regions for the Incongruent Versus Congruent Condition for the Typically Developing Group, and the Comparisons with the Attention-Deficit/Hyperactivity Disorder Group at the Pre-treatment and Post-treatment

Cortical regions	H	BA	Voxels	*Z* test	MNI coordinates		
					*x*	*y*	*z*
TD group							
Inferior frontal gyrus^a^	R	47	739	3.97	50	24	−6
Inferior frontal gyrus^b^	L	47	481	3.10	−56	20	2
Superior temporal gyrus	R	40	21	2.95	60	−46	20
Anterior cingulate gyrus	R	32	16	2.78	2	34	−8
TD vs. ADHD group							
TD>ADHD (pre-treatment)							
Superior temporal gyrus	R	40	248	3.14	58	−48	18
Inferior frontal gyrus^a^	R	47	35	3.10	36	24	−18
Angular gyrus	R	39	149	2.97	52	−64	32
Inferior temporal gyrus	R	22	14	2.86	62	−8	−18
Middle temporal gyrus	R	19	33	2.75	52	−64	10
Dorsal anterior cingulate cortex^c^	R	9	778	2.28	6	44	−2
Dorsolateral prefrontal cortex^c^	R	9	123	2.21	20	56	36
ADHD (pre-treatment)>TD							
Postcentral gyrus	L	2	318	3.56	−48	−22	50
Supplementary motor area	L	6	743	3.34	6	−8	66
Supplementary motor area	R			3.17	−8	−8	60
Inferior parietal lobule	L	40	403	3.22	−34	−46	52
TD>ADHD (post-treatment)							
Postcentral gyrus	R	3	574	2.97	12	−36	64
Postcentral gyrus	L	3	223	2.83	−26	−42	60
Inferior parietal lobule	L	40	18	2.70	−34	−40	46
ADHD (post-treatment)>TD							
Superior frontal gyrus	R	9	46	2.78	30	50	30
Inferior frontal gyrus^c^	R	47	58	2.74	30	28	−16

Coordinates of activation peak(s) within a region based on a *z* test are given in the MNI stereotactic space (*x, y, z*). All reported regions were *p* < 0.005 uncorrected with a voxel size larger than or equal to 10.

^a^ *p* < 0.05 for FWE corrected with the use of an anatomical mask.

^b^ *p* < 0.10 for FWE corrected with the use of an anatomical mask.

^c^ *p* < 0.05 uncorrected with a voxel size larger than or equal to 10.

ADHD, attention-deficit/hyperactivity disorder; BA, Brodmann's area; FWE, family-wise error; H, hemisphere, L, left; MNI, Montreal Neurological Institute; R, right; TD, typical developing.

Significant differences at a lower threshold were also revealed in the dACC and the rDLPFC between the ADHD group in the pre-treatment and the TD group (Table 3). In the post-treatment, no significant difference in the rIFG, dACC, or rDLPFC was found in the ADHD group relative to the TD group. And thus, treatment with ORADUR-methylphenidate might normalize the brain activations in the rIFG, dACC, and rDLPFC.

### Correlations between brain activation and neuropsychological performance

To conduct the correlations between the changes in neuropsychological performances and the changes in brain activation, we first built anatomical masks from the WFU PickAtlas toolbox for three ROIs, including the right IFG, right DLPFC, and dACC. We extracted beta value (signal intensity) for these ROIs in the ADHD group at pre-and post-treatment. We then correlated these beta values with neuropsychological performances, partialing out ADHD symptoms (i.e., CGI-ADHD-S, DSM Criteria-INATT, and DSM Criteria-HYPER) covariates.

Table 4 manifests the results of correlation analyses. Regarding correlations with measures of RVP, the changes of brain activation in the rIFG (*r* = 0.451, *p* = 0.023, Supplementary Fig. S4a) and the dACC (*r* = 0.477, *p* = 0.016, Supplementary Fig. S4b) were positively correlated with the increase of A′. Regarding correlations with measures of CCPT, the changes of both the rIFG (*r* = −0.446, *p* = 0.026, Supplementary Fig. S4c) and dACC (*r* = −0.447, *p* = 0.025, Supplementary Fig. S4d) were negatively correlated with the change of response style.

**Table 4.** Increasing Brain Activation with the Change of Neuropsychological Performances Between Pre-treatment and Post-treatment, Controlling for Attention-Deficit/Hyperactivity Disorder Symptoms

	Partial correlation coefficient	*p*
RVP		
Increase with A′ (target sensitivity)		
rIFG	**0.451**	**0.023** ^*^
rDLPFC	0.108	0.608
dACC	**0.477**	**0.016** ^*^
Increase with the probability of hit		
rIFG	0.359	0.078
rDLPFC	0.331	0.106
dACC	0.255	0.219
CCPT		
Increase with response style		
rIFG	**−0.446**	**0.026** ^*^
rDLPFC	−0.266	0.200
dACC	**−0.447**	**0.025** ^*^
Increase with perseveration		
rIFG	−0.268	0.195
rDLPFC	**−0.465**	**0.019** ^*^
dACC	−0.187	0.370

Bold values indicate *p*-values less than 0.05.

^*^ *p-*value <0.05.

ADHD, attention-deficit/hyperactivity disorder; CCPT, Conners' Continuous Performance Test; dACC, dorsal anterior cingulate cortex; rDLPFC, right dorsolateral prefrontal cortex; rIFG, right inferior frontal gyrus; RVP, Rapid Visual Information Processing.

Moreover, there was a negative correlation between the activation change of the rDLPFC and the increase of perseveration (*r* = −0.465, *p* = 0.019, Supplementary Fig. S4e).

## Discussion

The present study aimed at examining the therapeutic effects of 8-week treatment with a new drug of methylphenidate, ORADUR-methylphenidate, on drug-naive children with ADHD using fMRI. We found that ORADUR-methylphenidate increased activation in the dACC, rIFG, and rDLPFC. However, the activation of these brain regions increased differently. ORADUR-methylphenidate upregulated activations in the dACC and rDLPFC in the ADHD group and normalized the activation difference in the rIFG between the ADHD and TD groups.

In addition, increased activation in the dACC was positively correlated with the improvement in focused attention, measured by the response style of the CCPT. Increased activation in the rIFG was correlated with the improvement in inhibitory control and focused attention, measured by the A′ of the RVP and response style of the CCPT, respectively. Increased activation in the rDLPFC might be correlated with the reduction in cognitive flexibility, measured by increased perseveration of the CCPT.

Hypoactivation in the dACC has been associated with the deficits of cognitive processes in patients with ADHD. For example, lower activity was found in drug-naive subjects with ADHD during reward processing (Carmona et al. 2012). Our previous work found that the dACC was essential in suppressing inappropriate responses during the counting Stroop task (Chou et al. 2015; Fan et al. 2018).

Upregulated dACC activation after treatment with psychostimulant may imply cognitive processing involvement that could enhance capability for decision making (Schweren et al. 2017). In addition, previous studies have shown that improvement in cognitive functioning after treatment with methylphenidate might be mediated by enhancement of salience associated with greater task focus (ter Huurne et al. 2015), consistent with our findings of the correlation between increased activation in the dACC and the improvement in focused attention.

The DLPFC plays an essential role in the cognitive processes of attention (Curtis and D'Esposito 2003) and impulsivity control (Cho et al. 2010). Previous fMRI studies have found hypoactivation in the right DLPFC during selective and sustained attention tasks in patients with ADHD (Hart et al. 2013). A recent fMRI study showed that a single clinical dose of methylphenidate could enhance the activations in the DLPFC during a sustained attention task in drug-naive adolescents with ADHD (Kowalczyk et al. 2019). Besides, long-term treatment with methylphenidate enhances DLPFC activations during an interference inhibition task (Bush et al. 2008).

The present study showed that methylphenidate might reduce the cognitive flexibility of children with ADHD measured by the perseveration of CCPT, with a significant correlation between the increased activation of the DLPFC and the decrease of perseveration. Previous studies have demonstrated that dopamine (Ang et al. 2018) and noradrenaline (Alexander et al. 2007) played an important role in modulating the cognitive processes of flexibility and perseveration. Administration of methylphenidate was associated with a reduction in cognitive flexibility in healthy subjects (Fallon et al. 2017). Our findings suggested that methylphenidate might influence cognitive flexibility by modulating the DLPFC networks in children with ADHD.

Meta-analyses of fMRI studies have shown that the right IFG was a crucial brain region for inhibitory control and attentional processing (Wager et al. 2004; Simmonds et al. 2008). Dysfunction in the right IFG has been observed across fMRI studies on ADHD (Cortese et al. 2012). After treatment with ORADUR-methylphenidate, the present study found normalized activation in the right IFG in children with ADHD and improved inhibitory control and focused attention.

Several fMRI studies on the treatment effect of methylphenidate have also demonstrated increased activation in the IFG relative to placebo during response inhibition and attention tasks (Rubia et al. 2009a; Cubillo et al. 2014). A meta-analysis of 14 fMRI datasets in children with ADHD reported that the increased activation of the right IFG was the most consistent effect of methylphenidate (Rubia et al. 2014). The evidence combining the results from the meta-analysis and ours strongly supports that the increase and normalization of brain activations in the right IFG is one of the major biomarkers for the clinical effect of methylphenidate on ADHD.

Our previous study found that the osmotic release oral system-methylphenidate could increase activation in the IFG without significant change in the dACC and DLPFC (Chou et al. 2015), which was partially consistent with the findings of the present study. The discrepancy might be accounted for by several factors, including types of once-a-daily methylphenidate formulations and treatment durations.

There are some methodological limitations to be considered in the present study. First, given the lack of a placebo group, it could not be determined whether the improvements in behavioral symptoms and neuropsychological functions might be accounted for by the maturity or placebo effect. Second, our results of the neurobiological effects of ORADUR-methylphenidate may not be generalized to other methylphenidate formulations.

Third, the present study participants were recruited from only one medical center in Taiwan, and thus replication with different populations of individuals with ADHD was warranted. Fourth, due to the sample's moderate size, our present study might result in limited power to show the effects of ORADUR-methylphenidate on functional brain activations.

Therefore, future studies with larger samples were needed to validate our findings. Fifth, given that all the participants with ADHD had no comorbid psychiatric conditions based on the strict inclusion and exclusion criteria, our results might not be generalized to other ADHD populations.

Several methodological features of our present study constitute its strengths, despite these preceding limitations. First, drug-naive patients with ADHD were recruited to avoid potential confounding pharmacological effects on functional brain activations. Second, our fMRI study incorporating task designs (counting Stroop) inside the scanner and neuropsychological testing (RVP, CCPT) outside the scanner allowed us to examine the pharmacological mechanisms of methylphenidate for improving specific neurocognitive processes of focused attention, impulsivity, and inhibition control in children with ADHD.

## Conclusions

This is the first study investigating the neural correlates of improved neuropsychological functions after 8-week treatment with ORADUR-methylphenidate in drug-naive children with ADHD. We found that the effect of ORADUR-methylphenidate is most prominent on the rDLPFC and dACC, which are associated with improving impulsivity and focused attention. In addition, ORADUR-methylphenidate may also normalize the activation of the rIFG, enhancing focused attention and inhibitory control.

## Clinical Significance

Our findings may expand the understanding of the effects of methylphenidate on cognitive control via the fronto-cingular network (Salehinejad et al. 2021), providing an insight into the underlying neurophysiological process of methylphenidate response in children with ADHD.

## Supplementary Material

Supplemental data

Supplemental data

Supplemental data

Supplemental data

Supplemental data

Supplemental data

Supplemental data
