# Supplementary material for: A Counting Stroop Functional Magnetic Resonance Imaging Study on the Effects of ORADUR-Methylphenidate in Drug-Naive Children with Attention-Deficit/Hyperactivity Disorder
Source: J Child Adolesc Psychopharmacol. 2022 Nov 15;32(9):467–75. doi: 10.1089/cap.2022.0024 (PMC9700368; doi:10.1089/cap.2022.0024)

**Supplementary Figure 1**.

Examples of the congruent, incongruent, and control conditions in the counting Stroop task in Chinese.


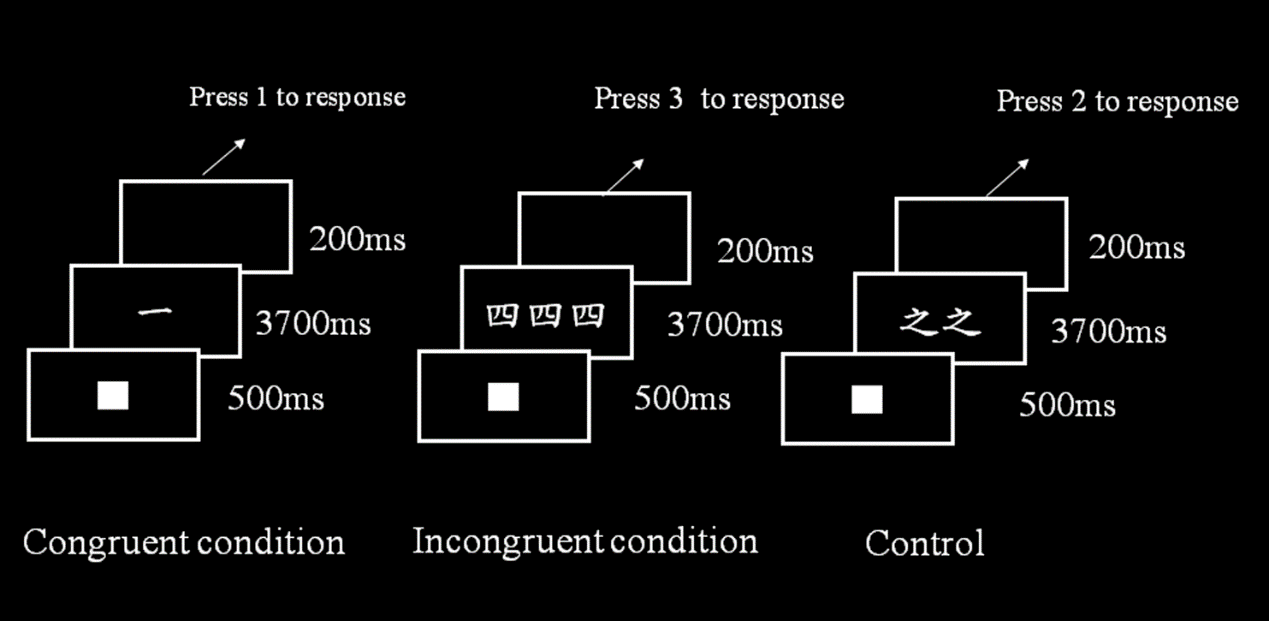

Supplement: Supplemental data [file Suppl_FigS1.doc]
