# Supplementary material for: A Counting Stroop Functional Magnetic Resonance Imaging Study on the Effects of ORADUR-Methylphenidate in Drug-Naive Children with Attention-Deficit/Hyperactivity Disorder
Source: J Child Adolesc Psychopharmacol. 2022 Nov 15;32(9):467–75. doi: 10.1089/cap.2022.0024 (PMC9700368; doi:10.1089/cap.2022.0024)
Supplement: Supplemental data [file Suppl_TableS2.doc]

**Supplementary Table 2**

*Means and standard deviations of realignment parameters for the ADHD Group at pre-and post-treatment.*

| **Realignment parameter** | **Pre-treatment** | **Post-treatment** | ***p*-value** |
| --- | --- | --- | --- |
| **Translation (mm)** | | | |
| *X-axis* | 0.24 (0.32) | -0.13 (0.13) | .112 |
| *Y-axis* | 0.36 (0.33) | -0.42 (0.45) | .584 |
| *Z-axis* | 0.44 (0.44) | 0.44 (0.53) | .997 |
| **Rotation (degree)** | | | |
| *Pitch* | <0.01 (0.01) | <0.01 (0.01) | .696 |
| *Roll* | <0.01 (0.01) | <0.01 (0.01) | .680 |
| *Yow* | <0.01 (0.01) | <0.01 (0.01) | .288 |

ADHD, attention-deficit/hyperactivity disorder.
