# Supplementary material for: A Counting Stroop Functional Magnetic Resonance Imaging Study on the Effects of ORADUR-Methylphenidate in Drug-Naive Children with Attention-Deficit/Hyperactivity Disorder
Source: J Child Adolesc Psychopharmacol. 2022 Nov 15;32(9):467–75. doi: 10.1089/cap.2022.0024 (PMC9700368; doi:10.1089/cap.2022.0024)
Supplement: Supplemental data [file Suppl_FigS2.doc]

**Supplementary Figure 2**.

Flowchart of the study procedure.


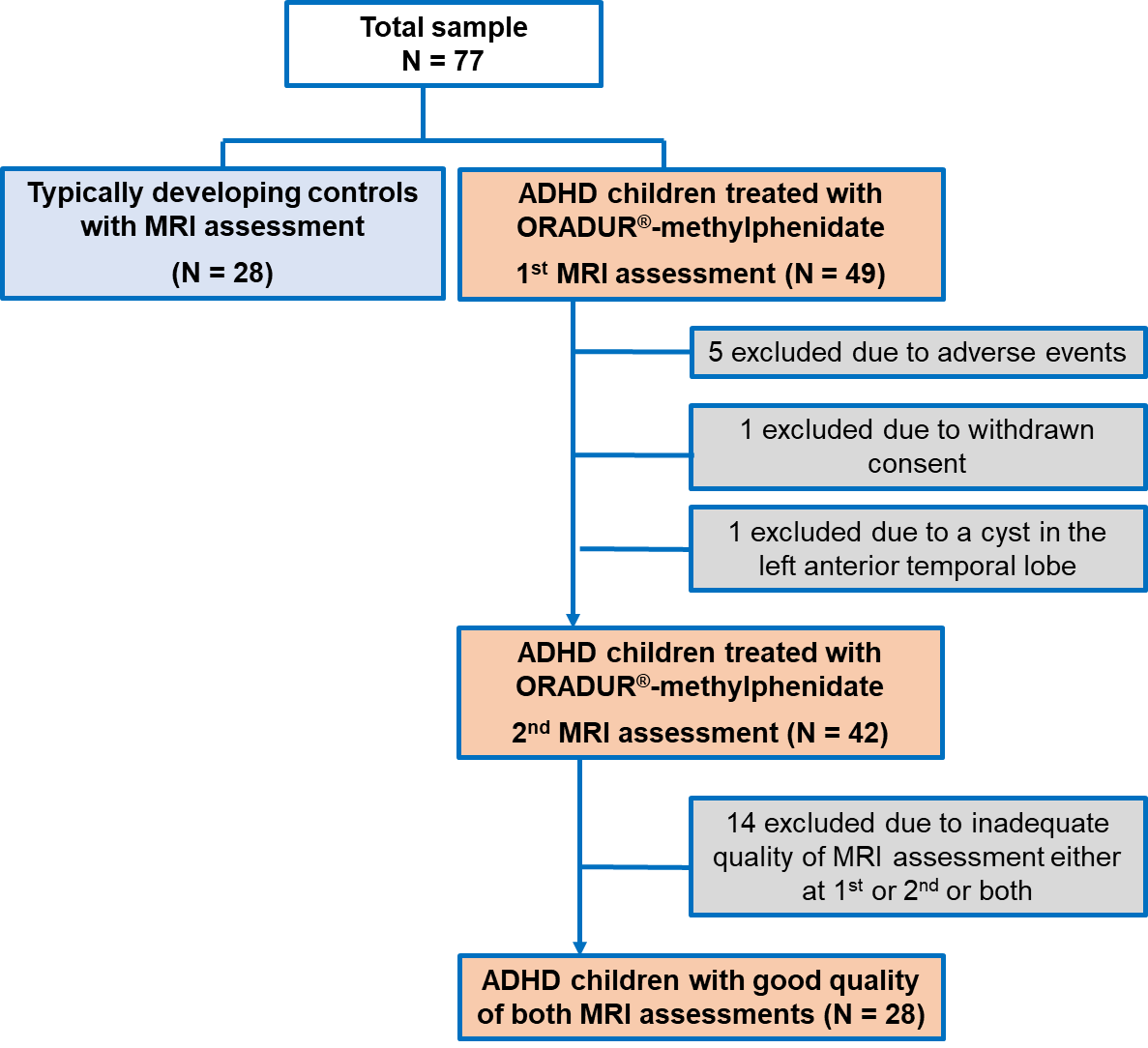


ADHD = attention-deficit/hyperactivity disorder
