# Supplementary material for: A Counting Stroop Functional Magnetic Resonance Imaging Study on the Effects of ORADUR-Methylphenidate in Drug-Naive Children with Attention-Deficit/Hyperactivity Disorder
Source: J Child Adolesc Psychopharmacol. 2022 Nov 15;32(9):467–75. doi: 10.1089/cap.2022.0024 (PMC9700368; doi:10.1089/cap.2022.0024)
Supplement: Supplemental data [file Suppl_TableS1.doc]

**Supplementary Table 1**

Demographic characteristics of the ADHD and TD groups.

|  | **ADHD (n=28)** | | **TD  (n=28)** | | ***t-*Statistics** | ***p* value** |
| --- | --- | --- | --- | --- | --- | --- |
| **Sex (Male/Female)** | 22/6 | | 22/6 | |  |  |
| **Age Range** | 7-13 | | 8-16 | |  |  |
| **Age, mean (SD)** | 10.29 | (2.17) | 11.35 | (2.61) | 1.87 | 0.066 |
| **Full-Scale IQ, mean (SD)** | 99.29 | (10.56) | 104.39 | (11.78) | 1.65 | 0.105 |

ADHD, attention-deficit/hyperactivity disorder; TD, typically developing controls.
