# Supplementary material for: A Counting Stroop Functional Magnetic Resonance Imaging Study on the Effects of ORADUR-Methylphenidate in Drug-Naive Children with Attention-Deficit/Hyperactivity Disorder
Source: J Child Adolesc Psychopharmacol. 2022 Nov 15;32(9):467–75. doi: 10.1089/cap.2022.0024 (PMC9700368; doi:10.1089/cap.2022.0024)
Supplement: Supplemental data [file Suppl_TableS3.doc]

**Supplementary Table 3**

The behavioral performance on the counting Stroop task in three conditions for the ADHD group at the pre-treatment and the post-treatment, and the TD group.

|  | **ADHD group** | | | |  | |
| --- | --- | --- | --- | --- | --- | --- |
|  | **Pre-treatment** | | **Post-treatment** | | **TD group** | |
| Reaction time (ms) |  | |  | |  | |
| Congruent | 1151 | (253) | 1088 | (194) | 1003 | (280) |
| Incongruent | 1207 | (294) | 1103 | (212) | 1079 | (274) |
| Control | 1126 | (239) | 1051 | (180) | 1005 | (267) |
| Accuracy (%) |  |  |  |  |  |  |
| Congruent | 97 | (5) | 95 | (10) | 97 | (6) |
| Incongruent | 93 | (8) | 93 | (8) | 95 | (6) |
| Control | 97 | (3) | 94 | (9) | 97 | (5) |

ADHD, attention-deficit/hyperactivity disorder; TD, typically developing controls
